# Designing a Smartphone-Based Pulse Oximeter for Children in South Africa (Phefumla Project): Qualitative Analysis of Human-Centered Design Workshops With Health Care Workers

**DOI:** 10.2196/54983

**Published:** 2024-05-30

**Authors:** Elif I Ilhan, Lucia N Jola, Marieke M van der Zalm, Mike Bernstein, Pierre Goussard, Andrew Redfern, Anneke C Hesseling, Graeme Hoddinott, Eric D McCollum, Carina King

**Affiliations:** 1 Desmond Tutu TB Centre, Department of Paediatrics and Child Health, Faculty of Medicine and Health Sciences, Stellenbosch University, Cape Town, South Africa; 2 PhysioMonitor, San Roman, CA, United States; 3 Department of Paediatrics and Child Health, Faculty of Medicine and Health Sciences, Stellenbosch University, Cape Town, South Africa; 4 School of Public Health, Faculty of Medicine and Health, The University of Sydney, Sydney, Australia; 5 Global Program for Pediatric Respiratory Sciences, Eudowood Division of Pediatric Respiratory Sciences, Johns Hopkins School of Medicine, Baltimore, MD, United States; 6 Department of Global Public Health, Karolinska Institutet, Stockholm, Sweden

**Keywords:** pediatrics, human-centered design, participatory design, pulse oximeter, South Africa, smartphone, mobile phone

## Abstract

**Background:**

Pulse oximeters noninvasively measure blood oxygen levels, but these devices have rarely been designed for low-resource settings and are inconsistently available at outpatient clinics.

**Objective:**

The *Phefumla* project aims to develop and validate a pediatric smartphone-based pulse oximeter designed specifically for this context. We present the process of human-centered oximeter design with health care workers in South Africa.

**Methods:**

We purposively sampled 19 health care workers from 5 clinics in Khayelitsha, Cape Town. Using a human-centered design approach*,* we conducted participatory workshops with four activities with health care workers: (1) they received 3D-printed prototypes of potential oximeter designs to provide feedback; (2) we demonstrated on dolls how they would use the novel oximeter; (3) they used pile sorting to rank design features and suggest additional features they desired; and (4) they designed their preferred user interface using a whiteboard, marker, and magnetized features that could be repositioned. We audio recorded the workshops, photographed outputs, and took detailed field notes. Analysis involved iterative review of these data to describe preferences, identify key design updates, and provide modifications.

**Results:**

Participants expressed a positive sentiment toward the idea of a smartphone pulse oximeter and suggested that a pediatric device would address an important gap in outpatient care. Specifically, participants expressed a preference for the prototype that they felt enabled more diversity in the way it could be used. There was a strong tendency to prioritize pragmatic design features, such as robustness, which was largely dictated by health care worker context. They also added features that would allow the oximeter device to serve other clinical functions in addition to oxygen saturation measurement, such as temperature and respiratory rate measurements.

**Conclusions:**

Our end user–centered rapid participatory approach led to tangible design changes and prompted design discussions that the team had not previously considered. Overall, health care workers prioritized pragmatism for pediatric pulse oximeter device design.

## Introduction

Hypoxemia, defined as an abnormally low peripheral arterial oxyhemoglobin saturation (SpO_2_) of <90%, is an important risk factor for death among children with lower respiratory infections in low- and middle-income countries (LMICs) [1-4]. An estimated 7 million children were hospitalized with hypoxemic pneumonia in 2019, and in sub-Saharan Africa, 28% (95% CI 25%-35%) of children with acute respiratory diseases were hypoxemic [5]. During outpatient care, the burden of hypoxemia may be considerable, with 2019 estimates suggesting a 23.1% prevalence among children with respiratory illnesses [5]. Pulse oximeters are medical devices that noninvasively measure SpO_2_ and can therefore detect hypoxemia.

Frequent pulse oximeter use is associated with positive health outcomes such as reducing mortality rates and has been found to be cost-effective in low-resource settings [1,6]. Although oximeters are commonly used in pediatric clinical care in high-income countries, they are not consistently available in LMICs [4], especially during outpatient care where most children first access care and their illness may be more treatment responsive. The COVID-19 pandemic led to large investments being made into oxygen ecosystems, including pulse oximetry [7]; however, it did not focus on overcoming key implementation challenges for children. Pediatric pulse oximetry implementation in LMICs is restricted by barriers such as cost, lack of appropriately designed pediatric devices and probes, disruptive movements of small children, unavailability of devices, lack of training and supervision, lack of maintenance, lack of electricity, and health care provider misconceptions [8-14]. A pediatric-specialized, low-cost, smartphone-based pulse oximeter device could potentially address many of these implementation barriers and serve as a valuable tool in outpatient LMIC settings.

The *Phefumla* project aims to cocreate a low-cost, smartphone-based, reflectance pulse oximeter device for children that is optimized for LMIC outpatient contexts. Reflectance oximetry, unlike transmittance oximetry, measures the relative ratio of unabsorbed red and infrared light that is reflected off of tissues rather than through tissues to produce an estimate of SpO_2_ [15]. A key source of inequities in health is access to diagnostic services, with almost half of the global population having little or no access to diagnostics [16]. Part of this inequality stems from devices designed for high-income and inpatient settings that are cost-prohibitive to purchase, sustain consumables, and maintain. To address this challenge, there is a need for a holistic framework to guide the design of medical devices so that they may be contextually appropriate for the settings in which they will be used [17].

We used a human-centered design (HCD) approach, with the aim of achieving a contextually appropriate device that meets the specific health care needs of the population [18]. The HCD method is one of many approaches to co-design and was chosen for this study given its successful application in previous global health intervention and medical device development projects [19-25]. In this paper, we describe the participatory HCD processes with health care workers (HCWs) and how this led to design changes, as an example of a rapid approach to medical device development that centers inclusion.

## Methods

### Overview

**Figure 1.**
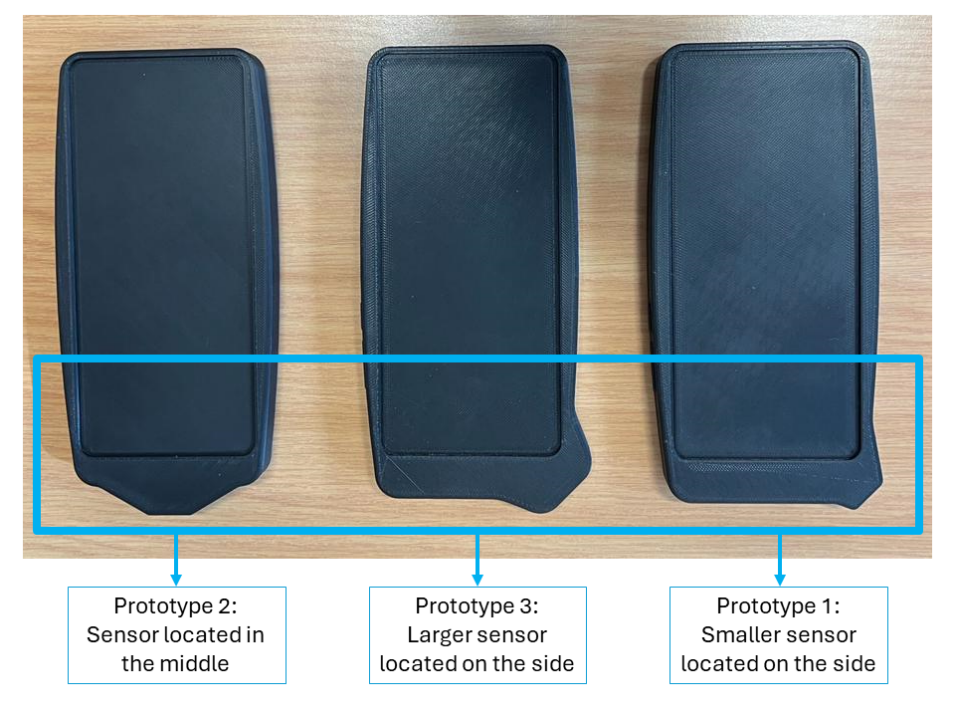
3D-printed *Phefumla* reflectance oximeter prototypes.

We conducted a qualitative observation study of participatory workshops that drew on the HCD approach, with HCWs in Khayelitsha, Cape Town, South Africa, from September 1-16, 2022. For these workshops, we had 3D-printed 3 prototype reflectance devices, all based on the same smartphone model being housed inside a case that would contain the oximeter sensor and additional hardware for processing (Figure 1). These prototypes were developed by the *Phefumla* team to prompt HCW reflections on the size and positioning of the sensor while keeping all other factors consistent.

### Setting

The East subdistrict of Khayelitsha is a low-income and low-resource area in Cape Town, South Africa, often referred to as a township. It has an estimated population of 450,000 people [26], who are predominantly Black African (99%) and the majority of whom live in informal housing [27]. First-tier primary health care (PHC) in South Africa is provided primarily through nurse-led clinics and community health centers, which are available within 5 km of 90% of the population and is often the first point of contact [28]. These facilities are free of cost and provide comprehensive basic services such as maternal, child, and reproductive health; HIV and tuberculosis testing and treatment; and care for noncommunicable diseases and common ailments [28]. Secondary care is delivered at district hospitals, which conduct minor procedures, and the third tier consists of tertiary hospitals that have the infrastructure, specialists, and equipment for major surgeries [29]. Many obstacles limit adequate implementation of health services at the PHC level in South Africa, including the HIV/AIDs pandemic, shortages of HCWs, unequal distribution of resources, and the legacy of the apartheid era [30]. This study was conducted at PHCs.

### HCD Approach

**Figure 2.**
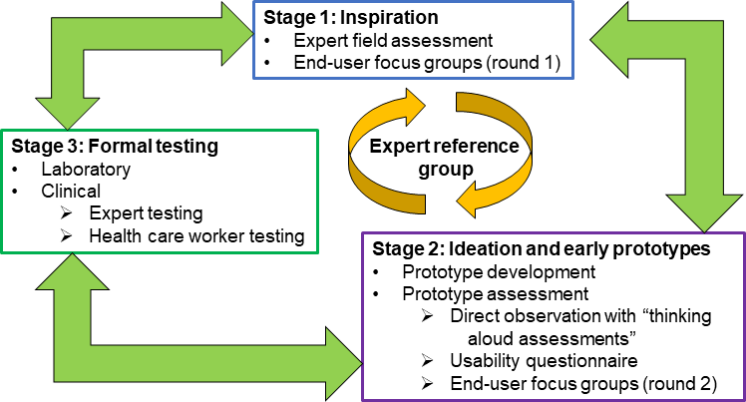
The human-centered design approach.

HCD is based on using “techniques which communicate, interact, empathize and stimulate the people involved, obtaining an understanding of their needs, desires and experiences which often transcends that which the people themselves actually realized” [31]. This approach encourages stakeholders, experts, and end users—in our case, HCWs—to generate knowledge collaboratively to co-design a medical device [32]. Through involving end users in the design process, HCD allows for the development of devices that are locally and contextually appropriate and can meet the specific health care needs of the population [18]. The key principles of HCD are the active involvement of users and a clear understanding of user and task requirements; iteration of design solutions, where end users provide feedback on design solutions starting early in the process; and making use of multidisciplinary design teams [33]. The HCD approach consists of three iterative stages: (1) inspiration, (2) ideation and prototyping, and (3) formal testing. In this study, we report activities conducted in stage 2, the ideation and prototyping of the *Phefumla* smartphone oximeter development (Figure 2). This builds on our stage-1 findings that explored HCWs’ current experience with pediatric pulse oximetry, which will be published elsewhere.

### Participatory Workshops

We conducted participatory workshops, consisting of 4 activities, to facilitate the process of having HCWs co-design a smartphone-based reflectance oximeter (Table 1). The discussion guide is available in Multimedia Appendix 1.

**Table 1.** Summary of participatory design workshop activities.

Activities	Resources and tools	Description
1. Design preference	Three 3D-printed plastic prototypes of potential device designs	Participants were given 3 different rapid prototypes and asked questions about the devices’ design. These included how they felt about the placement of the sensor on the device; how confident they felt placing the device on a child for a reading; how easy they believe the device would be to clean; and how durable the device was. Participants were encouraged to give suggestions and state preferences.
2. Device use	Two dolls (1 infant sized and 1 slightly larger sized)	Participants were asked to demonstrate how they would use the 3 prototypes on a child, specifically where they would place the sensor for taking a measurement on 2 different sized dolls to represent younger and older infants.
3. Feature ranking	A pile of cards with design features written on them	Participants were asked to rank 11 design features, which were deemed important from stage 1 of the HCD^a^ process (eg, battery life), from most to least important. They were also given an opportunity to add their own features on blank cards with markers.
4. User interface	A magnetic board and magnets of different design features of the interface (SpO_2_^b^ reading, waveform, pulse, bouncing bar, buttons, charging symbols, date, and time)	Participants were asked to arrange interface components as they would like the screen of the device to look. They were provided with a whiteboard marker to include any other features.

a HCD: human-centered design.

b SpO_2_: oxyhemoglobin saturation.

We conducted a pilot design workshop with 3 research nurses from the Desmond Tutu TB Centre to check the quality and coherency of the planned activities. Following the relevant consenting procedures, 1-hour-long design workshops were held with 7 small groups of our sample’s HCWs (2-4 HCWs per group). These workshops were conducted by 2 female postgraduate research assistants with comprehensive knowledge and experience of qualitative data collection (EII and LNJ) under the supervision and with the assistance of a pediatric pulmonologist (EDM). Small groups were chosen for pragmatic reasons to minimize disruption to clinical service at the facilities and were conducted in the clinics.

### Sampling and Participants

Participants were sampled from a larger pool of participants who had taken part in the previous stage of the study. Stage 1 of the HCD process (inspiration) involved small group discussions with HCWs focusing on barriers and challenges to routine pediatric oximetry use. These HCWs were therefore primed before the co-design workshops to think about the pros and cons of pulse oximeter features. Five clinics in the East subdistrict of Khayelitsha were eligible, and HCWs were purposefully sampled (rich case) using the following inclusion criteria: (1) having experience taking pulse oximeter measurements in children and (2) having taken part in the previous stage of the *Phefumla* study. Participants who had consented in stage 1 to be contacted were followed up to setup face-to-face meetings. Participants were given a small monetary voucher (worth approximately US $15) and provided with refreshments as reimbursement for their time.

### Data Collection

Data were collected via audio recordings and photographs taken of activity end results, as well as through comprehensive observation notes. A semistructured workshop guide was developed and used in English, the predominant working language in health care settings in South Africa. However, most participants were native Xhosa speakers, and some discussions were held in Xhosa; LNJ is a native Xhosa speaker and acted as a translator for these sections. The 2 researchers who facilitated the workshops alternated between (1) asking questions and leading facilitation and (2) keeping detailed field notes.

### Analysis

Data were analyzed using the framework of exploratory qualitative analysis. Exploratory research is concerned with exploring a phenomenon more deeply to gain a granular understanding of it and has 2 key aspects: open-mindedness and flexibility [34]. Recordings were repeatedly listened to by the 2 researchers who conducted the workshops (EII and LNJ), alongside looking through the captured pictures and written field notes. The wider research team had preidentified key design features of interest based on a rapid scoping review, stage-1 small group discussions, and team expertise. Quotes and notes taken during the workshops were mapped together by EII and LNJ under these categories of design features, using Microsoft Excel. This initial mapping framework was shared and discussed with the entire research team, where findings were discussed and probed. This was done iteratively until the team decided on actionable feedback for the pulse oximeter prototype and shared them with the engineer (MB).

### Ethical Considerations

Approval was obtained from the City of Cape Town to recruit HCWs and conduct the project at 7 clinics in the East subdistrict of Khayelitsha. Institutional approval was obtained from Johns Hopkins University (IRB00294436), Stellenbosch University (N22/01/009), and the Swedish Ethics Board (Dnr 2022-01897-01). Facility managers and other relevant gatekeepers were approached after receiving approval to ask for permission to access HCWs. All participants provided written, informed consent. Field notes were anonymized and did not record any identifiable data from HCWs, and recordings were stored in secure local servers to safeguard participant information.

## Results

### Overview

A total of 7 workshops were conducted with 19 HCWs. The most common reasons for participants from stage 1 not taking part in these stage-2 workshop were being ill, on leave, or absent at the clinic on the scheduled days; having been rotated to a different PHC; or having resigned. All 19 participants retained were nursing staff (including a range of nursing cadres), with 18 (95%) female participants and 1 (5%) male participant.

### Activity 1: Selecting a Preferred Prototype

Three 3D-printed prototypes were presented to the groups (Figure 1). The strongest preference was shown for prototype 2 with the sensor in the middle, with 4 (57%) of the 7 groups reaching a consensus on preferring this design. However, this was not unanimous, with 1 (14%) group preferring prototype 1, one (14%) group preferring prototype 3, and 1 (14%) group wanting a combination of prototype 3’s larger sensor size with prototype 2’s sensor location.

Participants primarily liked prototype 2 because of its sensor location being in the middle, noting that the device would be easier to use on a child as you would not have to angle it to get a reading, it did not matter if you were right- or left-handed, and some participants liked the larger size of the overall device and smaller size of the sensor (compared to the larger sensor of prototype 3). There were some concerns that the device itself was too large and that a smaller device would be easier to use, as well as concerns about having to hold the device without dropping it.

It’s easier for me to get grasp of the monitor and put it on the child, rather than using the corner.

[HCW, clinic 3]

When discussing the sensor, prototype 3’s larger sensor elicited a range of responses, with some HCWs stating that it would be too difficult to use on an infant or young child (eg “it’s too big”), whereas others thought the larger sensor size was a benefit, for example:

Very much easy [to use] because the sensor is bigger.

[HCW, clinic 1]

The sensor is nice and big.

[HCW, clinic 5]

Overall, participants displayed a positive sentiment to this style of device being easy to use on a child, and most participants felt comfortable placing the sensor correctly on a child. Robustness was a concern in several groups, as it was noted that a smartphone screen can break when dropped, and participants offered several modifications in relation to this:

It would be better if it were rubber or had a pouch, so it does not break.

[HCW, clinic 1]

The back must be rubbery, and the outer part is rubbery too.

[HCW, clinic 3]

If it is a glass screen it will [break easily].

[HCW, clinic 4]

All groups stated the device would be very easy to clean, with the most common suggestion for cleaning the device being wiping it with a disinfectant and cloth after each use. All groups felt it would be easy to store as well, with suggestions such as to keep it in a locked drawer, cabinet, or room or to include a storage pouch with the device:

Important that it’s got a pouch—a bag, so it doesn’t get too dusty.

[HCW, clinic 1]

### Activity 2: Using the Device on a Child

For activity 2, we asked participants to indicate for each prototype where they would take the pulse oximeter measurements on an infant-sized doll and a larger toddler-sized doll. The purpose was to understand how this novel device would be instinctively applied. The most common location of measurements for infants included the sole of foot, followed by the palm of hand, the hand, the thumb, and toes. These were similar for the older toddler–sized doll, with the sole of foot also being preferred, although HCWs noted that toddlers can kick. Infrequent answers included the wrist, the chest, the forehead, and the neck, which a participant noted would be beneficial as it would not require a child to be undressed. We deliberately did not prompt HCWs to consider specific locations, and it is likely that HCWs defaulted to appendages (ie, hands and feet) that are the most commonly used with a standard pulse oximeter, even if the positioning on those appendages (eg, the palm of the hand) differed.

### Activity 3: Design Feature Selection

Table 2 present the results from the feature pile sorting activity, showing the features considered as the 5 most important among the groups. When asked to elaborate on their ranking, participants stated that they first considered what would be essential for the device to function (eg, battery lasting) and that the rest were add-ons (eg, apps installed) that would be nice but were not necessary for core functioning. There was a strong preference displayed for pragmatism in this context:

The ones on top are the most important because they’re going to sustain the device.

[HCW, clinic 3]

**Table 2.** Features ranked among the top 5 for each group.

Feature	Groups (n=7), n (%)	Example quotations for prioritizing features
Portable device	7 (100)	“You can take it anywhere, for example if it is needed in emergency...then you can take it there.” (HCW^a^, clinic 4) “So you can take the sick baby to another room and take the device to the next room” (HCW, clinic 2) “Because we have three triages in this clinic” (HCW, clinic 3)
Does not break when dropped	6 (86)	“We are working with kids. It’s inevitable that it will fall. It is important that it doesn’t break easily when it falls. The kids might not want it and push it away from them and then it falls.” (HCW, clinic 4) “Because we are designing for a small baby not an adult so there’s high chances of it falling” (HCW, clinic 1) “Maybe you’re gonna be busy with an emergency so you’re going to be scared so you’re gonna be shivering or shaking maybe, and the baby will also be fighting you, so at least if it drops it mustn’t break easily” (HCW, clinic 2)
Long battery life	6 (86)	“We’re seeing more than 30 children a day and sometimes we don’t have time to charge—there’s no break when they come. They start to come as early as half past 7 to 4 o’clock so there’s no time to say we’re still waiting for the battery to get full.” (HCW, clinic 4) “If there’s no battery, there’s no device.” (HCW, clinic 1) “Loadshedding [of electricity] is happening so it must be charged, and the battery must last” (HCW, clinic 2)
Can measure different parts of the body	5 (71)	“It doesn’t limit you so you can use it on whatever part of the body that you want” (HCW, clinic 2) “We’ve got limited sites where you can do accurate readings” (HCW, clinic 1)
Easy to clean	4 (57)	“Hygiene is very important because we’re dealing with kids, so if it’s easy to clean then it’s more safe.” (HCW, clinic 4) “Because it’s used between many patients” (HCW, clinic 5)

a HCW: health care worker.

We asked if there were any disagreements in the group, but all groups indicated that they were happy with the consensus reached after discussion.

Participants added the following tangible features: small size, protective cover and storage bag, device holder or stand, and time stamp. However, more generic statements such as “easy to use” and “user friendly” were also added. Some added distractions to the child, such as a colorful screen or pictures. In 1 group, the HCWs also added the inclusion of a probe—the key design feature we were proposing to move away from.

Participants who included “other apps installed” in their overall ranking of design features were asked which apps they desired. The predominant suggestion was to have an app that could also measure temperature and respiratory rate, with a high preference for a multimodal device being displayed. Other app suggestions included an app for referral to hospital emergency wards (known as the *Vula* mobile app in this setting) [35] and an app that referred participants to the emergency medicine practice guidelines [36]. However, there were mixed feelings toward additional apps, and these often were not ranked among the most important features, with the exception of 1 (14%) of the 7 groups. Some of reasons given were that other apps would not be used; that they would negatively affect the battery life of the device; or that people would use the apps for personal reasons. As some HCWs noted:

We’re not gonna use other apps.

[HCW, clinic 4]

I wonder if it’s not gonna affect the battery life.

[HCW, clinic 3]

People overuse it for personal things.

[HCW, clinic 2]

### Activity 4: Interface Design

For the user-interface design activity, participants tended to place pulse and SpO_2_ readings together (6/7, 86% groups), although there was variability in where on the screen these were placed as well as variability in the size of the icons. Further, 6 (86%) out of the 7 groups included both the waveform as well as the bouncing bar, with the following reasons: if one is not working, the other will; each feature gives you different information; and it makes the device more accessible in the case someone is only familiar with either the bouncing bar or the waveform. The majority (5/7, 71%) of the groups included icons indicating temperature and respiratory rate, further indicating their preference for a multimodal device. Two (29%) groups added a distraction for the child, such as a moving video with sound. The battery was mostly placed at the top of the screen so that HCWs could immediately see whether the device needed to be charged when switched on. When asked what alarms and sounds were wanted, the main preference was for a sound when there was an abnormal reading. Furthermore, the preference was for a loud volume given the noisy environment of the clinics.

## Discussion

In this study, we conducted design workshops with South African HCWs to develop a novel, pediatric-specialized pulse oximeter device, to ensure the device is context appropriate. Through the design workshops, we found that HCWs displayed an overall positive and enthusiastic sentiment toward such a device, seeing its value in clinical use with children with hypoxemia. The findings from these workshops were used to select oximeter prototype 2 (Figure 1), with the sensor in the middle, to take forward into the prototype testing stage, with key updates to the robustness and planned user-interface incorporated.

Participants displayed the strongest preference for a device design with a sensor in the middle, feeling that it was overall the easiest to work with. Although participants felt that a smartphone device would be easy to use on a child, clean, and store and felt confident in placing the sensor correctly, some had concerns over the robustness of the device. They provided multiple suggestions to overcome this, such as a pouch, case, or rubber casing, and as a result, we increased the robustness of the device to be able to resist a drop test. However, this may point toward a potential limitation in using a smartphone interface, which HCWs are largely familiar with and have likely had experiences of breakages. This prompted a discussion within the study team on whether the smartphone inside the casing could be replaced with a locally available phone, allowing for a more sustainable repair solution than most traditional medical devices. Although this was not dealt with at this stage of design, the HCW feedback triggered us to reflect on this aspect of the device in more depth and to plan for future prototypes.

We received the least in-depth feedback on the mock placement and use of the prototypes with dolls of infants. One issue may have been the design of the activity, using infant dolls to prompt discussion. As a key challenge in pediatric oximetry, as noted by the HCWs as well, is the children’s movement and them becoming agitated with measurements being done, using a real child may have resulted in more reflective responses. The locations that the HCWs largely defaulted to were the thumb, toes, hands, and feet—where oximeter probes are generally used currently, although not with the same versatility. We had hypothesized that a benefit of reflectance oximetry is the range of locations that could be used, which reduces both the HCWs’ need to disturb the child and restrict their movement (eg, their forehead or upper back).

Pragmatic concerns arose most strongly during the activity where design features were ranked. These findings speak to the context in which HCWs in LMICs work, where having usable, durable, and long-lasting devices is of the essence—with participants noting that once devices break, they are unlikely to be replaced. This is due to factors such as limited technical and biomedical support and ties into other literature regarding “medical equipment graveyards”—composed of obsolete or otherwise broken biomedical, donated equipment—which are a common occurrence across LMICs [37]. These findings also speak to similar findings in other literature, where opportunities for redesign in pulse oximeters in LMICs included similar themes such as battery charging and durability, probe fit, and sensitivity in pediatric populations [11].

Participants liked the idea of a multimodal device. Although there were various suggestions given for additional design features, a device that could take temperature and respiratory rate readings in addition to SpO_2_ was by far the most desirable design feature proposed. This was desirable to participants as one device with multiple modalities is pragmatically beneficial. This speaks to possible opportunities for integration in future device designs and further developments in the field of eHealth; however, this needs to be weighed against risks. There is the risk that more complex devices will be more expensive, have reduced usability, and not be optimized for the oximetry function. Therefore, the benefits need to be weighed against the added value of additional functions, as a device performing a core functionality well could be beneficial over a device that performs poorly across various functionalities [11,38].

Some of the themes raised by our participants were raised in other studies in LMICs, indicating a degree of generalizability. Khayelitsha is considered to be fairly representative of other low-resource, sub-Saharan African settings when considering HIV exposure, tuberculosis mortality rates, and quality of care. However, some contextual factors may be unique to a particular context. For example, Khayelitsha has access to electricity but frequently experiences power supply blackouts (loadshedding), which happens nationwide, meaning that mains-charged devices are acceptable but need a long battery life. In contrast, in other settings, solar-powered charging was prioritized as access to electricity in health facilities was not universal [11].

Our study has several strengths in being able to rapidly engage with a range of HCWs. However, we also had 3 key limitations. First, we came up with the initial idea for a reflectance pulse oximeter, hypothesizing that this could solve several usability issues for LMIC outpatient settings. Our participants were therefore restricted in their first prototypes to 1 type of oximeter that the research team had chosen. It is possible participants may have preferred an alternative design or traditional transmittance pulse oximeter. It may also have biased our team’s presentation of the device and interpretation of the data. However, the workshop researchers had no prior experience in oximetry and led the data collection and analysis process in an attempt to mitigate potential researcher bias. Second, given that our data collection and analysis process were designed to be rapid and pragmatic, we did not extensively pilot the instruments. Lastly, the workshops were conducted primarily in English. Clinical training is done in English and is the language spoken in most professional South African environments. However, it was not the majority of participants’ home or first language, which could be a potential limitation. We allowed participants to answer in whatever language they wanted to, and we always had a Xhosa-speaking researcher available to mitigate this limitation.

A contextually appropriate, low-cost, pediatric-specialized, smartphone-based reflectance pulse oximeter was seen to have potential clinical value in the South African context. The process of HCD allowed us to explore HCW’s design preferences qualitatively to design a prototype device that would address their specific needs. The overall preference was for a multimodal and pragmatic device, with our rapid participatory approach successfully leading to changes in the oximeter design executed by our engineer.

## Supplementary material

10.2196/54983 Multimedia Appendix 1 Design workshop discussion guide.

## Abbreviations

HCD: human-centered design
HCW: health care worker
LMIC: low- and middle-income country
PHC: primary health care
SpO_2_: oxyhemoglobin saturation

## References

[1] Lazzerini M, Sonego M, Pellegrin MC (2015). Hypoxaemia as a mortality risk factor in acute lower respiratory infections in children in low and middle-income countries: systematic review and meta-analysis. PLoS One.

[2] McCollum ED, King C, Ahmed S (2021). Defining hypoxaemia from pulse oximeter measurements of oxygen saturation in well children at low altitude in Bangladesh: an observational study. BMJ Open Respir Res.

[3] King C, McCollum ED (2020). Trends in the global burden of paediatric lower respiratory infections. Lancet Infect Dis.

[4] McCollum ED, King C, Colbourn T (2019). Pulse oximetry in paediatric primary care in low-income and middle-income countries. Lancet Respir Med.

[5] Rahman AE, Hossain AT, Nair H (2022). Prevalence of hypoxaemia in children with pneumonia in low-income and middle-income countries: a systematic review and meta-analysis. Lancet Glob Health.

[6] Lam F, Stegmuller A, Chou VB, Graham HR (2021). Oxygen systems strengthening as an intervention to prevent childhood deaths due to pneumonia in low-resource settings: systematic review, meta-analysis and cost-effectiveness. BMJ Glob Health.

[7] Badgujar KC, Badgujar AB, Dhangar DV, Badgujar VC (2020). Importance and use of pulse oximeter in COVID-19 pandemic: general factors affecting the sensitivity of pulse oximeter. Indian Chemical Engineer.

[8] Enoch AJ, English M, McGivern G, Shepperd S, Clinical Information Network (2019). Variability in the use of pulse oximeters with children in Kenyan hospitals: a mixed-methods analysis. PLoS Med.

[9] Ginsburg AS, Tawiah Agyemang C, Ambler G (2016). mPneumonia, an innovation for diagnosing and treating childhood pneumonia in low-resource settings: a feasibility, usability and acceptability study in Ghana. PLoS One.

[10] Graham HR, Bakare AA, Gray A (2018). Adoption of paediatric and neonatal pulse oximetry by 12 hospitals in Nigeria: a mixed-methods realist evaluation. BMJ Glob Health.

[11] King C, Boyd N, Walker I (2018). Opportunities and barriers in paediatric pulse oximetry for pneumonia in low-resource clinical settings: a qualitative evaluation from Malawi and Bangladesh. BMJ Open.

[12] Sheikh M, Ahmad H, Ibrahim R, Nisar I, Jehan F (2023). Pulse oximetry: why oxygen saturation is still not a part of standard pediatric guidelines in low-and-middle-income countries (LMICs). Pneumonia (Nathan).

[13] Zhang J, Tüshaus L, Nuño Martínez N (2018). Data integrity–based methodology and checklist for identifying implementation risks of physiological sensing in mobile health projects: quantitative and qualitative analysis. JMIR Mhealth Uhealth.

[14] Sarin E, Kumar A, Alwadhi V, Saboth P, Kumar H (2021). Experiences with use of a pulse oximeter multimodal device in outpatient management of children with acute respiratory infection during COVID pandemic. J Family Med Prim Care.

[15] Keogh BF, Kopotic RJ (2005). Recent findings in the use of reflectance oximetry: a critical review. Curr Opin Anaesthesiol.

[16] Fleming KA, Horton S, Wilson ML (2021). The Lancet Commission on diagnostics: transforming access to diagnostics. Lancet.

[17] Aranda-Jan CB, Jagtap S, Moultrie J (2016). Towards a framework for holistic contextual design for low-resource settings. International Journal of Design.

[18] Bartlett R, Boyle JA, Simons Smith J, Khan N, Robinson T, Ramaswamy R (2021). Evaluating human-centred design for public health: a case study on developing a healthcare app with refugee communities. Res Involv Engagem.

[19] Boyd N, King C, Walker IA (2018). Usability testing of a reusable pulse oximeter probe developed for health-care workers caring for children <5 years old in low-resource settings. Am J Trop Med Hyg.

[20] Bazzano AN, Martin J, Hicks E, Faughnan M, Murphy L (2017). Human-centred design in global health: a scoping review of applications and contexts. PLoS One.

[21] Fisher M, Johansen E (2020). Human-centered design for medical devices and diagnostics in global health. Global Health Innovation.

[22] Taylor Salisbury T, Atmore KH, Nhambongo I (2021). Integrating human-centred design into the development of an intervention to improve the mental wellbeing of young women in the perinatal period: the Catalyst project. BMC Pregnancy Childbirth.

[23] Asiedu A, Nelson AR, Gomez PP, Tappis H, Effah F, Allen C (2019). “It builds your confidence… you've done well”: healthcare workers' experiences of participating in a low-dose, high-frequency training to improve newborn survival on the day of birth in Ghana. Gates Open Res.

[24] Triplett NS, Munson S, Mbwayo A (2021). Applying human-centered design to maximize acceptability, feasibility, and usability of mobile technology supervision in Kenya: a mixed methods pilot study protocol. Implement Sci Commun.

[25] Liu C, Lee JH, Gupta AJ (2022). Cost-effectiveness analysis of human-centred design for global health interventions: a quantitative framework. BMJ Glob Health.

[26] (2020). Population data. Western Cape Government.

[27] (2013). City of Cape Town - 2011 Census suburb Khayelitsha. City of Cape Town.

[28] World Health Organization, Alliance for Health Policy and Systems Research (2017). Primary Health Care Systems (PRIMASYS)‎: case study from South Africa. World Health Organization.

[29] Lim J, van Loggerenberg E, Chater R (2016). Country profile: South Africa. Bertha Centre for Social Innovation & Entrepreneurship.

[30] Kautzky K, Tollman SM, Barron P, Roma-Reardon J (2008). South African Health Review 2008.

[31] Giacomin J (2014). What is human centred design?. Des J (Aldershot).

[32] Sandholdt CT, Cunningham J, Westendorp RGJ, Kristiansen M (2020). Towards inclusive healthcare delivery: potentials and challenges of human-centred design in health innovation processes to increase healthy aging. Int J Environ Res Public Health.

[33] Maguire M (2001). Methods to support human-centred design. Int J Hum Comput Stud.

[34] Stebbins RA (2001). Exploratory Research in the Social Sciences.

[35] Secure medical chat & patient referrals. Vula mobile.

[36] Practice guidelines. Emercency Medicine Society South Africa.

[37] Marks IH, Thomas H, Bakhet M, Fitzgerald E (2019). Medical equipment donation in low-resource settings: a review of the literature and guidelines for surgery and anaesthesia in low-income and middle-income countries. BMJ Glob Health.

[38] Baker K, Ward C, Maurel A (2021). Usability and acceptability of a multimodal respiratory rate and pulse oximeter device in case management of children with symptoms of pneumonia: a cross-sectional study in Ethiopia. Acta Paediatr.

