# Supplementary material for: Designing a Smartphone-Based Pulse Oximeter for Children in South Africa (Phefumla Project): Qualitative Analysis of Human-Centered Design Workshops With Health Care Workers
Source: JMIR Hum Factors. 2024 May 30;11:e54983. doi: 10.2196/54983 (PMC11154651; doi:10.2196/54983)
Supplement: Multimedia Appendix 1 [file humanfactors-v11-e54983-s001.docx]

**Appendix 1: Stage 2 Design workshop discussion prompts and participatory tools**

*Before you start, make sure you have all the equipment you will need, including a camera, and label the prototypes with numbers.*

Thank you for joining this workshop – we have taken the experiences and thoughts you shared with us previously and now want to get your input on the designs we came up with for the new smart-phone based oximeter. We will do four activities, and in each one, we want to know your preferences, your concerns, and suggestions for improvements – please elaborate on your reasons as much as possible.

1. Feedback on 3-D printed models

Looking at these prototypes, tell us what you think of this design? Please say the prototype number out loud each time, so we know which one you are referring to.

- How easy would this be to use on a child?
- Do you think this would be robust, or break easily?
- What do you think about placing the sensor? Would you feel comfortable placing it correctly?
- How easy would this be to keep clean and store?
- What do you think about the screen?

2. Location of measurements

*Provide the group with a newborn and an infant sized doll.*

Using these prototypes, can you demonstrate locations that would make sense to you to take a pulse oximetry measurement. We would like you to use the fingers and toes, but feel free to try other locations as well.

- What do you think would make this location good?
- What challenges do you think there will be taking the measurement in this location?

3. Feature pile sorting

*Provide the group with paper cards which each have one design feature that the oximeter could include, and some blank ones. Feature cards should include: long battery life; easy to clean; doesn’t break when being dropped; use in bright/low light; internet/Bluetooth connectivity; has other apps installed [ask to elaborate on which ones]; can measure different parts of the body; portable device; does not require a probe with a cord; spot check and continual monitoring mode; distraction for the child. Make sure you take a picture of the final order.*

Can you place these features in order of importance as a group? Were there any other features that you think are important? Feel free to use the spare cards to add more features.

- Why did you place them in this order?
- Thinking about the prototypes we just showed you – which do you think fits this priority list the best? Why?
- What changes do you think are needed to improve it further?
- Were there any disagreements in this order in the group?

4. Designing the interface

*Provide the group with an A3 print out of the phone screen, coloured pens, and print outs of: a bouncing bar; a waveform; numeric HR and SpO2 display; text feedback; start/stop button; settings/menu symbol; battery life symbol; date and time symbol; blank paper.*

Now we want to know what your preferences are for how the screen would look. Can you place these items onto the screen in your preferred arrangement as a group? Feel free to use the colour pens to add colour and the paper to add more features.

- Why did you place them in this way?
- Do you prefer the bar, waveform, or something else? Or all to be there together?
- Is the use of colour coding useful?
- What about sounds, are there any alarms or sounds you want to add?
- What changes do you think are needed to improve it further?
- Were there any disagreements in this design in the group?
